# Educating for health service reform: clinical learning, governance and capability – a case study protocol

**DOI:** 10.1186/s12912-016-0152-8

**Published:** 2016-05-27

**Authors:** Anne Gardner, Glenn Gardner, Fiona Coyer, Helen Gosby

**Affiliations:** School of Nursing, Midwifery and Paramedicine, Australian Catholic University, Canberra, ACT Australia; School of Nursing, Institute of Health and Biomedical Innovation, Queensland University of Technology, Brisbane, QLD Australia; Intensive Care Services, Royal Brisbane and Women’s Hospital, Brisbane, QLD Australia; Emergency Department, The Children’s Hospital at Westmead, Sydney, NSW Australia

**Keywords:** Nurse practitioner, Advanced clinical learning, Case study methods, Embedded case study, Capability

## Abstract

**Background:**

The nurse practitioner is a growing clinical role in Australia and internationally, with an expanded scope of practice including prescribing, referring and diagnosing. However, key gaps exist in nurse practitioner education regarding governance of specialty clinical learning and teaching. Specifically, there is no internationally accepted framework against which to measure the quality of clinical learning and teaching for advanced specialty practice.

**Methods:**

A case study design will be used to investigate educational governance and capability theory in nurse practitioner education. Nurse practitioner students, their clinical mentors and university academic staff, from an Australian university that offers an accredited nurse practitioner Master’s degree, will be invited to participate in the study.

Semi-structured interviews will be conducted with students and their respective clinical mentors and university academic staff to investigate learning objectives related to educational governance and attributes of capability learning. Limited demographic data on age, gender, specialty, education level and nature of the clinical healthcare learning site will also be collected. Episodes of nurse practitioner student specialty clinical learning will be observed and documentation from the students’ healthcare learning sites will be collected.

Descriptive statistics will be used to report age groups, areas of specialty and types of facilities where clinical learning and teaching is observed. Qualitative data from interviews, observations and student documents will be coded, aggregated and explored to inform a framework of educational governance, to confirm the existing capability framework and describe any additional characteristics of capability and capability learning.

**Discussion:**

This research has widespread significance and will contribute to ongoing development of the Australian health workforce. Stakeholders from industry and academic bodies will be involved in shaping the framework that guides the quality and governance of clinical learning and teaching in specialty nurse practitioner practice. Through developing standards for advanced clinical learning and teaching, and furthering understanding of capability theory for advanced healthcare practitioners, this research will contribute to evidence-based models of advanced specialty postgraduate education.

## Background

In the current fast-changing health service environment, innovative service models are being developed rapidly around the world. The fastest growing of these models in many countries is the nurse practitioner which has almost doubled in number over a short period in Australia [1, 2]. Nurse practitioners in Australia and elsewhere are specialist clinicians educated at Master’s level who work both in the hospital and community sectors. They work from a nursing model of care, enhanced by legislative support to prescribe, refer and diagnose. These activities enable the nurse practitioner to complete an episode of care without the need to ‘hand over’ the patient at critical points in the care trajectory, thereby providing continuity of care and improved patient outcomes. This study addresses key gaps in nurse practitioner education specifically in relation to practice, theory and governance (quality control) in clinical learning and teaching. This research is timely given the recently introduced Australian Tertiary Education Quality and Standards Agency Standards and similar changes internationally [3–6] and the focus on the quality and availability of clinical training by the National Health Workforce Taskforce in Australia. There is a clear need for research based specialty clinical practice standards for advanced practice education that will provide a framework for educational governance. This study will provide a theoretically informed clinical educational model to support learning and teaching for the nurse practitioner and other hybrid advanced clinical roles.

The aim of this study is:

To investigate educational governance of advanced clinical learning and teaching and its contribution to capability theory through exploration of specialty learning and teaching in a case study research context.

The specific objectives to be addressed are:

1. To explore issues of accountability and quality control between the clinical domain and the university in clinical, field-based specialty education.
2. To draw upon the findings from objective 1, to develop a theoretical framework of educational governance for field based specialty education.
3. For a purposeful sample of nurse practitioner students, to explore how a theoretical framework of capability influences curriculum learning outcomes.

### Literature review

The nurse practitioner is one in an increasing array of innovative service models in the health care workforce; others include the prescribing pharmacist [7, 8] and specialist physiotherapist [9]. Education of these new levels of clinician poses a unique challenge in that the roles cross traditional health discipline boundaries and consequently require a composite of skills, knowledge and expertise. Nurse practitioners manage complete illness episodes for patients and may be the only healthcare staff available, especially in regional or remote areas [10]. Research informed generic nurse practitioner standards were developed and implemented nationally in Australia in 2006 and recently updated [11, 12]. There has been strong support for the centrality of clinical learning as the optimum educational preparation with clinical rigour being maintained alongside the academic advantages of a Master’s degree from a tertiary institution [13]. A recent release of curriculum standards for nurse practitioner Masters’ degrees have confirmed the importance of dedicated clinical learning and teaching time and a move away from the apprenticeship system of training for advanced practice [11].

### Clinical learning and teaching models

In nursing as in other health disciplines with advanced practice roles, there are often conflicting pressures between the clinical learning environment and the institution conferring recognition of advanced specialty practice. For nurse practitioners the institution conferring recognition is the university although for other health professions it may be specialist organisations such as professional colleges [14]. Tensions arising from the contrasting clinical and academic requirements are sometimes hidden and all too rarely addressed successfully. Part of the solution to these tensions, arguably the key component, is an effective clinical learning and teaching model that addresses the complexity of advanced clinical education.

In terms of the complexity required, most undergraduate clinical learning and teaching models focus on competence attainment, but there are few well-developed models for postgraduate *advanced* clinical learning [15, 16]. Capability learning is one promising conceptual model for learning and teaching at postgraduate level that has been used for nurse practitioners and warrants further explication [13, 17–19]. A recent study conducted in collaboration with the Australian College of Nurse Practitioners (ACNP), identified a concise set of areas of advanced specialty practice or ‘metaspecialties’ for Australian nurse practitioners [20]. This proposed set is framed using contemporary language, informed by a national framework for nursing specialties [21] and previously identified specialty areas. The metaspecialties are: mental health care; aged and palliative care; care of people with long term conditions; emergency and acute care; child and family health care; and primary health care. These metaspecialties are not intended to be mutually exclusive and a nurse practitioner working within a particular scope of practice may need specialty standards from more than one metaspecialty.

### Educational governance

In clinical learning and teaching the issue of governance has received scant attention and yet is essential for quality and equity in clinical education. There is considerable research on development of beginner level competency in health related disciplines, specifically in relation to skill assessment, but few studies address the issue of accountability of clinical education [22] or the relationship between workplace learning and curriculum governance. This is particularly relevant in nurse practitioner education where, to date, governance for clinical education has primarily related to legal and contractual arrangements; with scant attention to standardisation and quality control of clinical learning across specialities in line with the requirements of an accredited curriculum. An effective clinical learning and teaching model that addresses the complexity of advanced clinical education and acknowledges the importance of strong governance is needed so that integrity of curriculum content is assured [20]. This governance or quality control has not, to date, been addressed conceptually or theoretically. A comprehensive framework for educational governance is urgently needed to ensure that the education needs of the student; the safety of the clinical facility and the curriculum standards of the education provider are to be met.

In summary, there is disquiet internationally and silence locally about the governance of advanced clinical education, especially where this education occurs remote from the auspices of the awarding tertiary institution but is recognised in the final degree testamur. This research will elucidate a framework for educational governance of advanced specialty clinical learning and teaching. This study will also build upon current capability research [23].

### Conceptual frameworks

Advanced and specialty clinical practice degrees are increasing in number at postgraduate level across many health disciplines to meet the healthcare needs of the community. While learning is concentrated in the clinical domain (the workplace) under the supervision of skilled professionals (clinical workplace supervisors or mentors), the qualification is awarded by universities. Moreover, clinical workplace supervisors or mentors may or may not have formal university affiliation and or postgraduate degrees or teaching qualifications. These conditions potentially raise issues of accountability and quality control between clinical domain and university. To bridge this possible divide, a governance framework would provide a more seamless approach to the assurance of quality control in advanced specialty clinical learning and teaching. An extensive review of the literature was undertaken to investigate discussion of both quality control and the utilisation of clinical competency standards in clinical learning and teaching for advanced specialty practice healthcare disciplines. Within the literature, scant discussion of educational governance in this context was evident. For the purpose of this study, our working definition of the term ‘educational governance’ encompasses evidence (or a lack thereof) for the curriculum integrity and quality control of advanced specialty clinical learning and teaching. A model of clinical learning and framework of education governance will be informed by the nurse practitioner standards framework, recently updated [11] and the model for nurse practitioner education [17]. The latter included an evidence based framework that emphasised the importance of clinically derived competencies (now more commonly referred to as standards at this advanced practice level) and incorporated a pedagogical approach to learning through the development of capability [11].

Capability learning provides the education model and will inform the development of a governance framework for learning and teaching of complex clinical skills and knowledge. Capability draws on the work of Schon [24] applying a constructionist approach and the notion of the reflective practicum or thinking in practice. Forneris argues that this understanding provides insight into how educators can help students to ‘create new knowledge and new action’ [25]. Capability has been used since the 1990’s in the context of understanding learning and teaching and to inform evaluation methodologies for practice in a range of professional occupations but scholarly dissemination of its application has been very limited until recently, primarily to its use in computer training [26]. Despite its value, capability has received very limited scholarly consideration in the field of education, in particular clinical education, where it can significantly scaffold knowledge generation. Its main application has been in the Trans-Tasman Nurse Practitioner Standards Project where the attributes of capability learning were all confirmed as being applied by nurse practitioners [13, 17, 18]. More recently it has been applied specifically in the area of emergency nurse practitioners [19]. The study will build on this recent extension of the application from generic standards to specialty clinical standards across several metaspecialties, focusing on generation of contemporary educational concepts and principles.

## Methods

An embedded case study design [27–30] will be used to identify the *case* (main unit of analysis), the *case boundary* (the limits of the case); the *context*, and the *data source* (sub-units of analysis). Informed by the above research objectives these design elements are as follows:

### The case

Specialty learning, educational governance and capability for advanced practice students in the clinical context.

### The case boundary

The case is bounded by nurse practitioner students’ clinical learning.

### The context

Health agencies where nurse practitioner student clinical learning takes place. This may be hospitals, community facilities or private residences.

### Sub-units

Within a purposeful sample of (up to 12) nurse practitioner students:

1. University academic staff (that is staff directly involved in liaison related to clinical learning and teaching of nurse practitioner students – role titles vary between universities but will be referred to as university academic staff in this study)
2. Clinical mentors
3. Occasions of nurse practitioner student clinical learning and teaching
4. Nurse practitioner students

The participating educational institution for the study will be one Australian university that offers an accredited nurse practitioner Master’s degree. Data will be collected relevant to the context and sub-units as follows:

1. ***Context***: Data collection will be conducted at the clinical learning context (health agency) of up to 12 nurse practitioner students.
2. ***Sub-Units***: For each nurse practitioner student, data will be collected from interviews with the student, university academic staff and clinical mentor. Additional data will be collected from direct observation of learning and teaching episodes; and review of relevant learning and teaching documents.

### Data collection methods

To meet the objectives the data collection methods are now described in more detail:

*Objective 1:* To explore issues of accountability and quality control between the clinical domain and the university in clinical, work-based specialty education.

- In-depth interviews will be conducted with consenting university academic staff supporting the nurse practitioner students’ clinical learning. The interviews will be guided by objectives related to educational governance. The semi-structured interviews will also gather demographic data including age, gender, specialty and education level.
- In-depth interviews will be conducted with consenting clinical mentors who are responsible for teaching nurse practitioner specialty standards. The interviews will be guided by objectives related to educational governance. The semi-structured interviews will also gather demographic data including age, gender, specialty, education level and the nature of the service where the clinical mentors are employed.
- Teaching documents associated with clinical learning will be reviewed for each nurse practitioner student participant. These documents may include but will not be limited to supervision or learning contracts, submitted assessments; other documents associated with clinical placements and associated feedback.
- Observational data will be collected from nurse practitioner students’ clinical learning and teaching episodes to identify factors that have the potential to influence quality of learning and teaching. Observation sessions will be negotiated with each participant depending upon supervision arrangements and clinical demands. Duration of observation sessions will vary between 30 min to 2 h.

*Objective 2:* For a purposeful sample of nurse practitioner students, to explore how a theoretical framework of capability influences curriculum learning outcomes.

- Observational data will be collected from nurse practitioner students’ clinical learning and teaching episodes to identify attributes of capability learning. Observation sessions will be negotiated with each participant depending upon supervision arrangements and clinical demands. Duration of observation sessions will vary between 30 min to 2 h.
- In-depth interviews will be conducted with participating nurse practitioner students. The interviews will be guided by objectives about learning experiences related to the attributes of capability. The semi-structured interviews will also gather demographic data including age, gender, specialty and education level.
- In-depth interviews will be conducted with consenting clinical mentors who are responsible for nurse practitioner specialty learning and teaching. The interviews will be guided by objectives related to capability learning.
- Teaching documents, as described above under Objective 1, are also likely to provide evidence for capability learning, because they are likely to include reflection and feedback.

These objectives and the linkage with data elements are also summarised in Table 1.

**Table 1:** Linkage between research objectives and data components

Objectives	Sub-units	Methods including data types
1. To explore issues of accountability and quality control between the clinical domain and the university, that is educational governance, in clinical, work-based specialty education.	University academic staff.	In-depth interviews with university academic staff supporting the nurse practitioner students’ clinical learning, especially perceptions of academic governance of specialty learning and teaching.
	Clinical mentors.	In-depth interviews with clinical mentors responsible for teaching nurse practitioner specialty standards, including perceptions of influences on clinical learning and teaching.
	Occasions of nurse practitioner student clinical learning and teaching.	Direct observation of nurse practitioner students’ clinical learning and teaching episodes.
	Nurse practitioner students.	In-depth interviews with nurse practitioner students, especially perceptions of influences on clinical learning and teaching and teaching documents associated with clinical learning for each nurse practitioner student.
2. For a purposeful sample of nurse practitioner students, to explore how a theoretical framework of capability influences curriculum learning outcomes.	Clinical mentors.	In-depth interviews with clinical mentors responsible for teaching nurse practitioner specialty standards, especially perceptions of meeting curriculum learning outcomes related to the attributes of capability.
	Nurse practitioner students.	In-depth interviews with nurse practitioner students, especially perceptions of meeting curriculum learning outcomes related to the attributes of capability.
	Occasions of nurse practitioner student clinical learning and teaching.	Observational data of nurse practitioner students’ clinical learning and teaching episodes. Teaching documents associated with clinical learning for each nurse practitioner student.

*Sample recruitment*: Following approval from relevant human research ethics committees, study information and consent packages will be disseminated to nurse practitioner students enrolled at the participating education institutions. Consenting students will be interviewed; agree to their university academic staff and clinical mentors being approached for interviews; be observed during clinical learning episodes and provide access to learning and teaching documents. The study research team will contact specific university academic staff and clinical mentors to seek consent for their participation once consent has been received from each nurse practitioner student.

### Data management and analysis

Limited demographic data will be managed using IBM SPSS Statistics 20. Descriptive statistics will be used to report age groups, areas of specialty and types of clinical facility where clinical learning and teaching is observed. Counts and percentages will be used to describe types of documents retrieved. Data will be aggregated to a level that ensures anonymity of individuals but informs transferability of findings beyond the specific case.

Data will be analysed to address each objective separately. Objective 1 will be addressed first. The analysis will be inductive because there are no existing educational governance frameworks. Analysis for objective 2 will be both inductive and deductive. Semi-structured interviews will be conducted by telephone, audio-recorded and transcribed. Qualitative data from interviews, observations and documents will be organised using QSR NVivo 10. Qualitative data will be analysed using processes of initial coding, aggregation of codes, identification of conceptual patterns and interpretive analysis of identified concepts. First each data type will be analysed separately up to the point of identification of conceptual patterns. Then triangulation will be undertaken to look for patterns across data types. Finally, identification of conceptual patterns across data types will inform a framework of educational governance including areas that need further exploration. Data will be explored to confirm the existing capability framework and data will also be explored to look for additional characteristics of capability and capability learning.

### Study status

At time of manuscript submission, all ethics approvals have been obtained, and recruitment of participants and data collection is in progress.

## Discussion

This national project has widespread significance and will contribute to ongoing development of the Australian health workforce. In terms of preparing the rapidly growing nurse practitioner workforce this research will have both national and international impact; there is no published information on evidence-based models of advanced specialty education. This research will deliver postgraduate educational models and guiding frameworks that are internationally relevant, for nursing as well as other health disciplines. This study brings together the relevant interface of the industry through the ACNP as the representative professional group and academics as the end-providers of degrees.

There will be two levels of outcome and impact from the study. At one level the outcomes will inform the health services through development of a framework of educational governance of complex clinical learning and teaching of advanced specialty practice for the universities offering Master of Nurse Practitioner courses and the body evaluating the curricula (the Australian Nursing and Midwifery Accreditation Council). At a higher level, the study outcomes will be an important contribution to the body of knowledge about models of clinical learning that go beyond competency attainment and expand understanding about capability and capability learning. The development of a framework for educational governance of complex clinical learning and teaching of advanced specialty practice will be instrumental in guiding and engendering debate in the context of interprofessional and multidisciplinary education and workplace learning where the growth of advanced specialty practice will ultimately take place.

## Abbreviations

ACNP: Australian College of Nurse Practitioners
